# The effect of time to neurosurgical or neuroradiological intervention therapy on outcomes and quality of care after traumatic brain injury, a registry-based observational study

**DOI:** 10.1186/s12245-024-00787-y

**Published:** 2024-12-20

**Authors:** Wasin Pansiritanachot, Sattha Riyapan, Sang Do Shin, Jirayu Chantanakomes, Netiporn Thirawattanasoot, Wichayada Rangabpai, Bongkot Somboonkul, Joo Jeong, Kyoung Jun Song, Wen-Chiu Chiang, Sabariah Faizah Jamaluddin, Kentaro Kajino

**Affiliations:** 1 Department of Emergency Medicine, Faculty of Medicine Siriraj Hospital, Mahidol University, 2 Wanglang Road, Siriraj, Bangkoknoi, Bangkok, 10700 Thailand; 2 Department of Emergency Medicine, Seoul National University College of Medicine, Seoul, South Korea; 3 Siriraj Emergency Medical Service Center, Faculty of Medicine Siriraj Hospital, Mahidol University, Bangkok, Thailand; 4 Department of Emergency Medicine, Seoul National University Bundang Hospital, Seongnam, Gyeonggi South Korea; 5 Laboratory of Emergency Medical Services, Seoul National University Hospital Biomedical Research Institute, Seoul, South Korea; 6 Department of Emergency Medicine, Seoul National University Hospital, Seoul, South Korea; 7 Department of Emergency Medicine, National Taiwan University Hospital Yunlin Branch, National Taiwan University College of Medicine, Taipei, Taiwan; 8 Department of Emergency Medicine, Faculty of Medicine, Universiti Teknologi MARA, Shah Alam, Selangor Darul Ehsan, Malaysia; 9 Traumatology and Critical Care Medical Center, National Hospital Organization Osaka National Hospital, Osaka, Japan

**Keywords:** Traumatic brain injury, Intervention, Mortality, Disability

## Abstract

**Background:**

Evidence regarding the effect of time to neurosurgical and neuroradiological intervention on outcomes in traumatic brain injury (TBI) across Asia-Pacific region is limited. This study evaluates the quality of care and outcomes for TBI patients undergoing neurosurgical and neuroradiological procedures at different timings.

**Methods:**

Adult TBI patients who received any neurosurgical or neuroradiological interventions during the year 2015–2022 in the Pan-Asian Trauma Outcome Study database were analyzed. The time to intervention, as the main exposure, was classified into three groups (Early, Intermediate, and Delayed) using Restricted Cubic Spline (RCS) analysis. The outcomes were in-hospital mortality and unfavorable neurological outcomes. W score was utilized to compare the quality of care among exposure groups. Multivariable logistic regression analysis and interaction analysis were performed to identify the association between the exposure groups and outcomes, reported as adjusted odds ratios (AOR) with 95% confidence intervals (CI).

**Results:**

A total of 1,780 patients were included. From the RCS analysis, patients were classified into three groups according to time to intervention: Early (< 1.9 h), Intermediate (1.9–4.1 h), and Delayed (> 4.1 h). According to the time to intervention, W score was − 8.6 in the early group, -1.1 in the intermediate group, and + 0.4 in the delayed group. Patients receiving intermediate and delayed intervention showed significantly lower mortality (AOR 0.64, 95% CI 0.47–0.86 and AOR 0.66, 95%CI 0.48–0.90, respectively).

**Conclusion:**

Early neurosurgical and neuroradiological interventions in TBI patients in the Asia-Pacific region were associated with lower quality of care and higher mortality. The quality of care should be focused and improved during the early hours of TBI.

**Supplementary Information:**

The online version contains supplementary material available at 10.1186/s12245-024-00787-y.

## Background

Trauma is among the leading causes of death and disability. Globally, 4.4 million annual deaths are attributed to traumatic injuries [1]. With the advancement in trauma care and emergency response systems, the relative contribution of death due to multiple organ dysfunction, acute respiratory distress syndrome, and sepsis has been decreasing dramatically over the past few decades. Traumatic brain injury (TBI) has become the leading cause of trauma-related death instead [2]. TBI accounted for 37% of all trauma-related deaths across European countries [3].

Timely definitive care is of the essence in TBI patients. Early craniotomy or hematoma drainage within 4 h of emergency department (ED) arrival significantly reduced mortality in TBI patients, according to a nationwide registry-based study [4]. A study also reported lower mortality when the time to craniectomy was within 5.3 h of injury in combat-related brain injury [5]. However, a recent meta-analysis questioned the universal efficacy of early surgical intervention in TBI patients [6]. It found that brain surgeries performed in the early period were unexpectedly linked to adverse outcomes, specifically in developing countries. Patients requiring immediate intervention were typically more complicated and higher in severity. Rushing to surgery might impede the resuscitation process, affecting the ‘quality of care’ in a real-life situation. This highlights the complexity of balancing timely care with the need for comprehensive treatment. Variations in EMS systems and healthcare disparities further complicate efforts to optimize TBI management and care quality. Evidence regarding the impact of prompt interventions on regional outcomes and various healthcare settings across the Asia-Pacific region remains limited.

Therefore, this study aimed to evaluate the quality of care and outcomes among TBI patients receiving neurosurgical and neuroradiological intervention at different timings across the Asia-Pacific region. Furthermore, the study examined how prehospital and interhospital transport settings might differently affect outcomes in TBI patients, emphasizing the need for tailored strategies to optimize TBI management in diverse systems.

## Methods

### Study design

This is a registry-based observational study using the Pan-Asian Trauma Outcome Study (PATOS) database. The manuscript adheres to the Strengthening the Reporting of Observational Studies in Epidemiology (STROBE) reporting guidelines [7].

### Study setting

PATOS is a large multinational emergency medical services (EMS)-based trauma registry network across the Asia-Pacific region, initiating data collection in 2015 [8]. The main purposes of PATOS were to benchmark emergency trauma care and improve survival outcomes in the Asia-Pacific region. The registry collected standardized data from 10 countries: India, Japan, Malaysia, Philippines, South Korea, Singapore, Taiwan, Thailand, United Arab Emirates, and Vietnam [9]. Trauma care systems, especially EMS systems, in Asia-Pacific countries were relatively new and underdeveloped compared to the systems in European countries and the United States.

The EMS systems varied among countries. Most of the countries had both Advanced Life Support (ALS) and Basic Life Support (BLS) teams [10]. Fire departments typically provided prehospital trauma care in countries like Korea, Japan, and Singapore, while hospital-based or community-based teams were common in Thailand, Malaysia, and the Philippines [11, 12]. The EMS team leaders were physicians in more than half of the participating sites. Nurses, emergency medical technicians (EMT), and paramedics were team leaders in Korea, Malaysia, Philippines, Singapore, and Taiwan [12].

Most participating sites were urban academic tertiary care hospitals, with a quarter designated as trauma centers [9]. Trauma teams were available in half of the participating sites. Licensed trauma surgeons were available in two-thirds of the participating sites [9].

On the national level, trauma care systems varied due to differences in health care infrastructure, resources, and policy priorities. National trauma triage protocols and patient transfer protocols existed in Korea and Japan [13]. Most participating sites generally followed Advanced Trauma Life Support (ATLS), with exceptions in Korea, Singapore, and Japan where national trauma guidelines existed [13].

### Study data source

The PATOS registry gathered data from 36 participating hospitals (27 tertiary, 8 secondary, and 1 primary hospital) across the Asia-Pacific region [9]. The registry collected trauma patients data of any severity who were transported by EMS ambulances in developed communities or non-EMS (non-professional) vehicles in developing communities to the emergency department of the participating hospitals [8].

To ensure data consistency and quality across participating sites, each site designated a research coordinator or principal investigator responsible for data collection, extraction, and input. The PATOS Data Quality Management Committee oversaw the central data cleaning process, conducted routine audits, and provided feedback to research teams to maintain high data quality standards. Additionally, regular meetings were held between the committee and site investigators to address inconsistencies and ensure adherence to standardized protocols [8].

### Population

This study included all adult (≥ 18 years) TBI patients who received any neurosurgical or neuroradiological interventions from every participating site during January 2015 to December 2022. The International Classification of Diseases 10th Edition (ICD-10) code S06 (intracranial injury) was used as an index for TBI patients. Neurosurgical and neuroradiological interventions were defined as the first recorded neurological operative procedures performed on the patients in the PATOS database, including both neurosurgical operations (such as craniectomy, craniotomy, and hematoma evacuation) and neuroradiological intervention (such as angioembolization) in the head region.

Patients were excluded if the primary outcome was missing. We also excluded patients whose time to intervention could not be measured. We also excluded patients with unknown systolic blood pressure.

### Variables and measurements

#### Exposure definition and measurement

The primary exposure, time to intervention, was defined as the interval between ED arrival and the initiation of the neurosurgical or neuroradiological intervention. In the latest guideline, the time of injury was used as a reference starting time [14]. However, in this study, we used ED arrival time instead for the following reasons: (1) the exact time of injury was likely inaccurate and missing in some cases, and (2) using ED arrival time as a starting point until the time of the surgery would directly reflect the effectiveness of in-hospital management.

The second exposure is the mode of transport. Prehospital transport was defined as the direct transportation of trauma patients from the scene to the ED. Interhospital transport was defined as the secondary transfer of trauma patients from another hospital.

#### Confounder definition and measurement

Confounder variables were categorized into 5 groups: general factors, injury factors, prehospital care, ED and hospital care, and injury severity. General factors included age, sex, and Charlson’s comorbidity index [15]. The injury factors included the intent of the injury (accidental, intentional), mechanisms of injury, place of injury, alcohol intake, and day of injury (weekday vs. weekend), and time of the injury. Prehospital care data included the top-level personnel, airway management, breathing & ventilation management, and fluid management. ED and hospital care data included vital signs, Glasgow Coma Scale (GCS), and types of intervention (neurosurgical versus neuroradiological intervention). For injury severity, we used the excess mortality ratio-based injury severity scale (EMR-ISS) which was a diagnosis-based injury severity scale for large data sets derived from the ICD-10 codes to depict injury severity [16].

### Outcome measures

The primary outcome was death, defined as in-hospital mortality. The secondary outcome was unfavorable neurological outcomes at discharge, defined as Glasgow Outcome Scale (GOS) 1–3 [17]. GOS is a 5-point scale score, categorized as (1) dead, (2) vegetative state, (3) severe disability, (4) moderate disability, and (5) good recovery [18]. This scale was chosen for its wide acceptance and standardized evaluation of functional recovery in TBI research.

### Statistical analyses

Confounding and outcomes variables were compared between exposure groups using median and interquartile range (IQR) for continuous variables, and numbers and percentages for categorical variables. Statistical significances were considered when the *p*-values were less than 0.05 using Wilcoxon sum rank test for continuous variables, and Chi-square test for categorical variables.

The categorization of time to intervention was determined using the Restricted Cubic Spline (RCS) analysis with four knots to model the non-linear relationship between time to intervention and mortality. Two key time points (1.9 and 4.1 h) were identified as knots where the relationship between time to intervention and mortality exhibited noticeable shifts, based on statistical analysis and visual inspection of the spline curve. The remaining two knots were placed at the extremes of the distribution to ensure adequate flexibility in fitting the model. Based on this analysis, patients were stratified into three groups according to time to intervention: early (< 1.9 h), intermediate (1.9–4.1 h), and delayed (> 4.1 h). These intervals not only reflect statistically significant inflection points but also aligned with practical clinical workflows in TBI management. The early group included cases requiring immediate intervention. In contrast, the delayed group represented interventions that occurred after stabilization, allowing for more comprehensive resuscitation, evaluation, or transfer. The intermediate group aligned with the critical therapeutic window frequently emphasized in TBI care, balancing timely intervention with adequate preparation.

For the main analysis, W score was also used to compare the difference in survival outcomes among three groups of patients: early, intermediate, and delayed interventions. W score is the difference between observed survivors and expected survivors per 100 patients [19]. The formula of the W score is (A-B)/(C/100). A is the actual number of survivors. B is the expected number of survivors based on the probability of survival (PS) from the Trauma and Injury Severity Score (TRISS) model which was derived from the Major Trauma Outcome Study (MTOS) in 1995 to predict survival and disabilities with coefficient revision in 2009 [20]. C is the total numbers of patients used for calculation of the PS. For example, a positive W score of + 2 indicates that there are 2 more survivors than predicted per 100 patients. Thus, W score represents the quality of the TBI care system within each group of patients. A positive W score indicates more survivors than predicted, reflecting superior care quality. A negative W score suggests fewer survivors than expected, potentially highlighting areas for improvement.

An additional analysis was performed using the multivariable logistic regression model. Potential confounding factors were tested and selected as confounders for the model when the *p*-value was less than 0.2 in univariate analysis between the exposures and factors. The association between exposure groups and outcomes was tested using multivariable logistic regression analysis and adjusted odds ratios (AOR) and 95% confidence interval (95% CI) were calculated from the model. We also compared the effect size of the time to intervention on the outcomes across the mode of transport in the final model as interaction terms.

#### Handling of missing data

Monotone logistic regression imputation was used to address missing data for key covariates, ensuring that the analysis included as many cases as possible while maintaining data integrity. The imputation model included patient demographics, injury severities and injury mechanisms as predictors to account for relationships among variables.

Critical variables such as primary outcomes and time-to-intervention were not imputed. Cases with missing values for these variables were excluded from the analysis to preserve the reliability and robustness of the results.

## Results

### Baseline characteristics

**Fig. 1:**
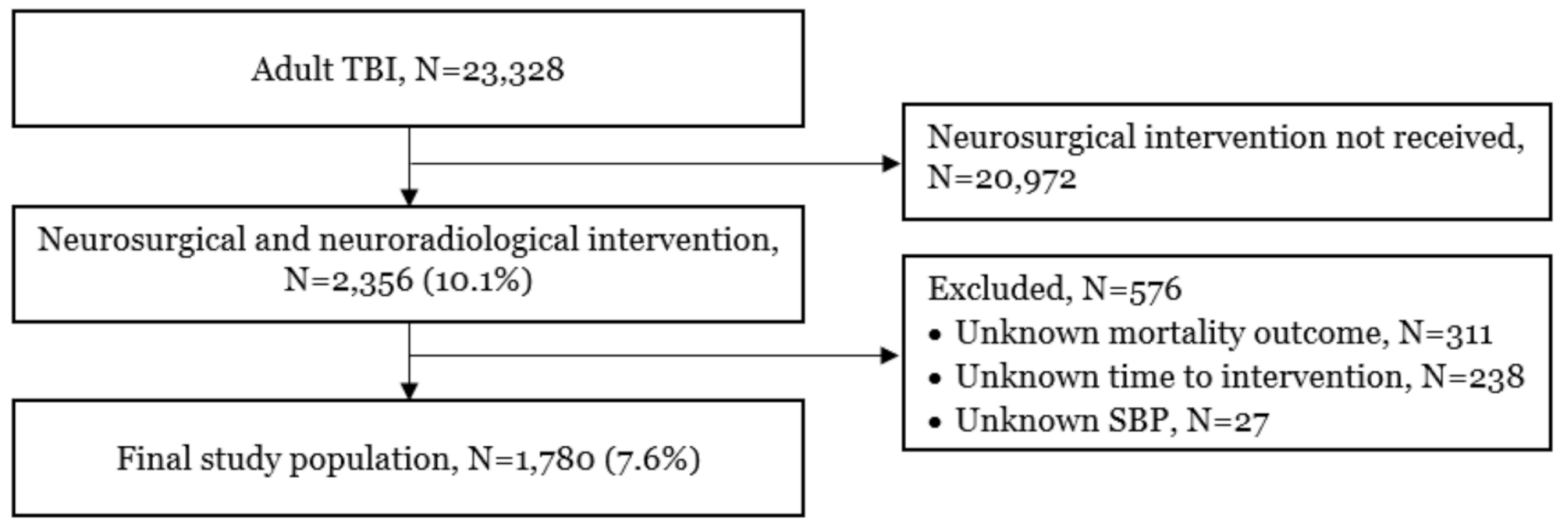
Study population TBI, traumatic brain injury SBP, systolic blood pressure

From 23,328 adult TBI patients during the study period, 2,356 (10.1%) patients received neurosurgical and neuroradiological interventions. A total of 576 patients were excluded; 311 for unknown mortality outcomes, 238 for unknown time to intervention, and 27 for unknown SBP. Ultimately, 1,780 patients were included in the final analyses (Fig. 1).

From the RCS analysis, the knots of 1.9 and 4.1 h were derived as cut-off time points. The median [IQR] time to intervention in each group was as follows: early 1.3 [1.0-1.6] hours, intermediate 2.7 [2.3–3.2] hours, and delayed 9.2 [5.7–25.3] hours after ED arrival.

Table 1 illustrates the baseline characteristics of patients according to time to intervention. There were 532 patients (29.9%) receiving early intervention, 541 patients (30.4%) receiving intermediate intervention, and 707 patients (39.7%) receiving delayed intervention. Most patients (98.5%) received neurosurgical operation, while a small number of patients (2.3%) received neuroradiological intervention. Regarding mortality, 36.1% of patients in the early group, 24.8% of patients in the intermediate group, and 18.3% of patients in the delayed intervention group died, respectively. Unfavorable neurological outcomes occurred in 65.2% of patients in the early group, 53.4% of patients in the intermediate group, and 43.6% of patients in the delayed intervention group.

**Table 1:** Demographic data according to time to neurosurgical and neuroradiological intervention

Variables		All		Time to Intervention						*P*-value
				Early (< 1.9 h)		Intermediate (1.9–4.1 h)		Delayed (> 4.1 h)		
		*N*	%	*N*	%	*N*	%	*N*	%	
All		1780	100.0	532	29.9	541	30.4	707	39.7	
Age group, years										0.003
	18–39	353	19.8	104	19.6	89	16.5	160	22.6	
	40–59	571	32.1	188	35.3	193	35.7	190	26.9	
	60–79	664	37.3	191	35.9	205	37.9	268	37.9	
	≥ 80	192	10.8	49	9.2	54	10.0	89	12.6	
Gender										0.402
	Male	1301	73.1	399	75.0	386	71.4	516	73.0	
	Female	479	26.9	133	25.0	155	28.7	191	27.0	
Charlson comorbidity index										0.006
	0	1390	78.1	423	79.5	419	77.5	548	77.5	
	1	114	6.4	28	5.3	32	5.9	54	7.6	
	2	156	8.8	60	11.3	44	8.1	52	7.4	
	≥ 3	120	6.7	21	4.0	46	8.5	53	7.5	
Injury intent										0.672
	Accidental	1741	97.8	518	97.4	531	98.2	692	97.9	
	Intentional	39	2.2	14	2.6	10	1.9	15	2.1	
Mechanism of injury										0.006
	Traffic accident	792	44.5	247	46.4	225	41.6	320	45.3	
	Fall	773	43.4	239	44.9	253	46.8	281	39.8	
	Collision	173	9.7	39	7.3	53	9.8	81	11.5	
	Others	42	2.4	7	1.3	10	1.9	25	3.5	
Place of injury										0.602
	Home	481	27.0	137	25.8	154	28.5	190	26.9	
	Public	1299	73.0	395	74.3	387	71.5	517	73.1	
Alcohol intake										0.065
	Yes	1371	77.0	391	73.5	422	78.0	558	78.9	
	No	409	23.0	141	26.5	119	22.0	149	21.1	
Day of injury										0.501
	Weekend	549	30.8	171	32.1	171	31.6	207	29.3	
	Weekday	1231	69.2	361	67.9	370	68.4	500	70.7	
Time of injury										< 0.001
	Day (7.00 AM − 6.59 PM)	1025	57.6	270	50.8	321	59.3	434	61.4	
	Night (7.00 PM − 6.59 AM)	755	42.4	262	49.3	220	40.7	273	38.6	
Mode of transport										< 0.001
	Prehospital transport	982	55.2	258	48.5	304	56.2	420	59.4	
	Interhospital transport	798	44.8	274	51.5	237	43.8	287	40.6	
Top-level personnel										< 0.001
	Physician	77	4.3	35	6.6	28	5.2	14	2.0	
	Nurse	353	19.8	136	25.6	108	20.0	109	15.4	
	EMT	1084	60.9	292	54.9	320	59.2	472	66.8	
	First responder	122	6.9	33	6.2	44	8.1	45	6.4	
	Unknown	144	8.1	36	6.8	41	7.6	67	9.5	
Airway management										0.046
	Advanced airway	27	1.5	8	1.5	9	1.7	10	1.4	
	Basic airway	72	4.0	23	4.3	26	4.8	23	3.3	
	No airway management	830	46.6	218	41.0	255	47.1	357	50.5	
	Unknown	851	47.8	283	53.2	251	46.4	317	44.8	
Ventilatory management										< 0.001
	Active ventilatory support	68	3.8	23	4.3	21	3.9	24	3.4	
	Passive ventilatory support	358	20.1	125	23.5	117	21.6	116	16.4	
	No ventilatory support	503	28.3	101	19.0	152	28.1	250	35.4	
	Unknown	851	47.8	283	53.2	251	46.4	317	44.8	
Intravenous fluid										0.056
	Yes	148	8.3	38	7.1	47	8.7	63	8.9	
	No	781	43.9	211	39.7	243	44.9	327	46.3	
	Unknown	851	47.8	283	53.2	251	46.4	317	44.8	
Systolic blood pressure, mmHg										0.104
	< 90	1676	94.2	496	93.2	504	93.2	676	95.6	
	≥ 90	104	5.8	36	6.8	37	6.8	31	4.4	
Heart rate, beats per minute										0.312
	< 60	122	6.9	44	8.3	33	6.1	45	6.4	
	60–100	1238	69.6	355	66.7	376	69.5	507	71.7	
	> 100	420	23.6	133	25.0	132	24.4	155	21.9	
Respiratory rate, rate per minute										0.808
	< 10	9	0.5	2	0.4	3	0.6	4	0.6	
	10–30	1728	97.1	514	96.6	525	97.0	689	97.5	
	> 30	43	2.4	16	3.0	13	2.4	14	2.0	
Glasgow coma scale										< 0.001
	3–8	657	36.9	298	56.0	210	38.8	149	21.1	
	9–12	292	16.4	70	13.2	100	18.5	122	17.3	
	13–15	663	37.3	117	22.0	168	31.1	378	53.5	
	Unknown	168	9.4	47	8.8	63	11.7	58	8.2	
Intervention										
	Neurosurgical	1754	98.5	520	97.7	536	99.1	698	98.7	0.166
	Neuroradiological	40	2.3	17	3.2	8	1.5	15	2.1	0.159
EMR-ISS										0.391
	0–24	93	5.2	36	6.8	23	4.3	34	4.8	
	25–44	1472	82.7	436	82.0	450	83.2	586	82.9	
	45–75	215	12.1	60	11.3	68	12.6	87	12.3	
**Outcomes**										
	Death	455	25.6	192	36.1	134	24.8	129	18.3	< 0.001
	Unfavorable neurological outcome	944	53.0	347	65.2	289	53.4	308	43.6	< 0.001

EMR-ISS, excess mortality ratio-adjusted injury severity score

Baseline characteristics of patients according to the mode of transport was illustrated in Supplementary Table S1. There were 982 patients (55.2%) in the prehospital group and 798 patients (44.8%) in the interhospital group. A significantly higher mortality in the prehospital group (29.4% vs. 20.8%, *p* < 0.001) was observed. Unfavorable neurological outcomes were comparable between groups (53.9% vs. 52.0%, *p* = 0.241).

### Main analysis (W score analysis)

W score was − 2.7 for overall patients, -5.0 for the subgroup of patients receiving prehospital transport, and + 0.1 for the subgroup of patients receiving interhospital transport, as shown in Table 2. According to the time to intervention, W score was lowest in patients receiving early intervention (early − 8.6, intermediate − 1.1, and delayed + 0.4).

**Table 2:** W score for overall patients and subgroups according to the mode of transport

Groups		Total (*N*)	Observed survival (*N*)	Expected survival (*N*)^1^	W score^2^
Overall					
	All	1780	1325	1373.7	-2.7
	Early	532	340	385.8	-8.6
	Intermediate	541	407	413.1	-1.1
	Delayed	707	578	574.8	0.4
Subgroup: Prehospital transport					
	All	982	693	742.2	-5.0
	Early	258	137	176.4	-15.3
	Intermediate	304	214	227.8	-4.5
	Delayed	420	342	338.0	1.0
Subgroup: Interhospital transport					
	All	798	632	631.5	0.1
	Early	274	203	209.4	-2.3
	Intermediate	237	193	185.2	3.3
	Delayed	287	236	236.9	-0.3

^1^ Expected survival was based on the probability of survival from the Trauma and Injury Severity Score (TRISS) prediction model

^2^ W scores is the difference between observed and expected survival rates per 100 patients. A positive W score indicates more survivors than predicted, while a negative W score suggests fewer survivor than predicted, reflecting poor quality of care

*Note*: Subgroup comparisons were selected to explore how time to neurological intervention and different transport modes impacted quality of care in patients with traumatic brain injury across the Asia-Pacific region

In the subgroup of patients receiving prehospital transport, W score was lowest among patients receiving early intervention (early − 15.3, intermediate − 4.5, and delayed + 1.0). In the subgroup of patients receiving interhospital transport, W score was also lowest among patients receiving early intervention (early − 2.3, intermediate + 3.3, delayed − 0.30).

### Additional analyses

Table 3 shows the results from the multivariable logistic regression analyses. After adjustment for confounders, patients receiving intermediate and delayed intervention had a significantly lower mortality compared to patients receiving early intervention (AOR 0.64, 95%CI 0.47–0.86 and AOR 0.66 95%CI 0.48–0.90, respectively). There was no significant difference in unfavorable neurological outcomes in the intermediate and delayed intervention groups (AOR 0.78, 95%CI 0.58–1.05 and AOR 0.86 95%CI 0.64–1.16, respectively). There was no difference in the rate of mortality (AOR 0.54, 95%CI 0.22–1.33) and unfavorable neurological outcome (AOR 1.17, 95%CI 0.52–2.64) between patients receiving prehospital transport and patients receiving interhospital transport.

**Table 3:** Multivariable logistic regression analysis for outcomes by time to neurosurgical and neuroradiological intervention and the mode of transport

Exposure	Outcome	Group	Total	Outcomes		Crude			Adjusted		
			*N*	*n*	%	OR	95% CI		AOR	95% CI	
Time to intervention											
	Death	Total	1780	455	25.6						
		Early	532	192	36.1	1.00			1.00		
		Intermediate	541	134	24.8	0.58	0.45	0.76	0.64	0.47	0.86
		Delayed	707	129	18.2	0.40	0.31	0.51	0.66	0.48	0.90
	Unfavorable neurological outcome	Total	1722	944	54.8						
		Early	529	347	65.6	1.00			1.00		
		Intermediate	524	289	55.2	0.65	0.50	0.83	0.78	0.58	1.05
		Delayed	669	308	46.0	0.45	0.35	0.57	0.86	0.64	1.16
Mode of transport											
	Death	Total	1780	455	25.6						
		Prehospital	982	289	29.4	1.00			1.00		
		Interhospital	798	166	20.8	0.63	0.51	0.78	0.54	0.22	1.33
	Unfavorable neurological outcome	Total	1722	944	54.8						
		Prehospital	943	529	56.1	1.00			1.00		
		Interhospital	779	415	53.3	0.892	0.74	1.08	1.17	0.52	2.64

OR, odds ratio; CI, confidence interval; AOR, adjusted odds ratio

The multivariable logistic regression model by time to neurosurgical intervention was adjusted for age, Charlson comorbidity index, mechanism of injury, alcohol intake, time of injury, mode of transport, top-level personnel, airway management, ventilatory management, intravenous fluid, systolic blood pressure, Glasgow coma scale score, and types of intervention

The multivariable logistic regression model by the mode of transport was adjusted for mechanism of injury, place of injury, alcohol intake, time of injury, top-level personnel, airway management, ventilatory management, intravenous fluid, systolic blood pressure, heart rate, Glasgow coma scale score, time to neurosurgical intervention, types of intervention and EMR-ISS

The interaction analysis showed marginally significant lower mortality only in patients in the intermediate intervention group receiving interhospital transport (AOR 0.84, 95%CI 0.70-1.00) (see Supplementary Table S2). There was no difference in mortality and unfavorable neurological outcomes according to time to intervention across the mode of transport in the other groups.

## Discussion

This study evaluated the quality of care for TBI patients undergoing neurosurgical and neuroradiological interventions at different timings and transport modes in the Asia-Pacific region. The highest mortality and excess mortality were observed in the early intervention group, while unfavorable neurological outcomes showed no significant variation across intervention timings or modes of transport. Notably, a marginal but significant lower mortality was identified in patients in the intermediate intervention group receiving interhospital transport.

The appropriate time to intervention and its impact on outcomes in TBI patients remains debated. Intensity and duration of elevated intracranial pressure were linked to poor outcomes, suggesting that prompt intervention should improve neurological recovery [21]. However, the recent meta-analysis proved otherwise, and aligned with the results of this study [6]. The higher mortality observed in the early intervention group was probably multifactorial. This group predominantly comprised patients with severe injuries necessitating immediate intervention, often presenting with critical conditions that limit the opportunity for thorough resuscitation and stabilization. In contrast, patients who survived long enough to receive intermediate or delayed interventions likely benefited from stabilization or might reflect a survival bias [22].

Systemic and logistical factors within the trauma care pathway may also contribute to these outcomes. Delivering high-quality emergency care within the critical early hours is particularly challenging in resource-variable settings. In parts of the Asia-Pacific region, limited prehospital stabilization, delays in imaging or surgical readiness, and resource constraints further exacerbate these challenges, leading to suboptimal outcomes for critically ill patients [12]. These findings emphasize the need for system improvements, rather than suggesting that immediate life-saving interventions should be avoided or delayed.

The W score analysis provides additional insight into the quality of trauma care. A negative W score, most prominent in the early intervention group, indicated ‘preventable deaths’ and reflected systemic deficiencies in prehospital and early in-hospital care [19]. These results underscore the urgency of measures to improve trauma care during the early hours of TBI care, especially in the prehospital setting where the W score was far more negative. Standardizing prehospital triage and resuscitation protocols across regions, enhancing the readiness of trauma teams, and streamlining in-hospital workflows, such as rapid imaging and operating room availability, may help mitigate early-phase care deficiencies [23–25]. Additionally, training programs for emergency care providers focused on managing high-severity TBI cases could improve care quality and outcomes for patients in the early intervention period. These measures should be endorsed internationally and adapted to the local EMS protocol.

### Limitation

This study has several limitations. First, despite rigorous quality control measures, data standardization inconsistencies across a multicenter registry persisted. For instance, variability in data sources—ranging from electronic medical records to direct patient surveillance—might affect the accuracy of neurological outcomes. Additionally, variables such as ED wait times and delays in surgical preparation were not captured, limiting the ability to fully assess in-hospital factors contributing to intervention timing and outcomes. Future studies should incorporate detailed prehospital and in-hospital metrics to better elucidate these relationships. Second, while logistic regression imputation was applied to handle missing data, this method did not account for potential unmeasured confounders. Alternative statistical approaches, such as propensity score matching or stratified analyses, could enhance comparability among intervention groups and should be considered in future studies. Lastly, the study utilized data from EMS systems in the Asia-Pacific region, which might differ significantly from those in other parts of the world. Variations in trauma care protocols and healthcare system capacities across regions may limit the generalizability of these findings to other settings.

## Conclusion

Early neurosurgical and neuroradiological interventions for the adult TBI patients in the Asia-Pacific region were associated with lower quality of care and higher mortality. Quality of care in the early hours of TBI should be focused and urgently improved. Risk factors related to higher mortality and disability should be investigated in the future study.

## Electronic supplementary material

Below is the link to the electronic supplementary material.

Supplementary Material 1

## Data Availability

The data that support the findings of this study are available from the PATOS study group. Restrictions apply to the availability of these data, which were used under license for this study, and so are not publicly available. Data are however available from the authors with the permission of the PATOS.
