# Supplementary material for: The effect of time to neurosurgical or neuroradiological intervention therapy on outcomes and quality of care after traumatic brain injury, a registry-based observational study
Source: Int J Emerg Med. 2024 Dec 20;17:193. doi: 10.1186/s12245-024-00787-y (PMC11660868; doi:10.1186/s12245-024-00787-y)
Supplement: Supplementary file 1 — Supplementary Material 1 [file 12245_2024_787_MOESM1_ESM.docx]

Table S1 Demographic data according to the mode of transport

| Variables | | All | | Mode of Transport | | | |  |
| --- | --- | --- | --- | --- | --- | --- | --- | --- |
|  |  |  |  | Prehospital transport | | Interhospital transport | | P-value |
|  |  | N | % | N | % | N | % |  |
| All | | 1780 | 100.0 | 982 | 55.2 | 798 | 44.8 |  |
| Age group, years | |  |  |  |  |  |  | 0.003 |
|  | 18-39 | 353 | 19.8 | 225 | 22.9 | 128 | 16.0 |  |
|  | 40-59 | 571 | 32.1 | 303 | 30.9 | 268 | 33.6 |  |
|  | 60-79 | 664 | 37.3 | 358 | 36.5 | 306 | 38.4 |  |
|  | ≥ 80 | 192 | 10.8 | 96 | 9.8 | 96 | 12.0 |  |
| Gender | |  |  |  |  |  |  | 0.647 |
|  | Male | 1301 | 73.1 | 722 | 73.5 | 579 | 72.6 |  |
|  | Female | 479 | 26.9 | 260 | 26.5 | 219 | 27.4 |  |
| Charlson comorbidity index | |  |  |  |  |  |  | 0.337 |
|  | 0 | 1390 | 78.1 | 782 | 79.6 | 608 | 76.2 |  |
|  | 1 | 114 | 6.4 | 59 | 6.0 | 55 | 6.9 |  |
|  | 2 | 156 | 8.8 | 82 | 8.4 | 74 | 9.3 |  |
|  | ≥ 3 | 120 | 6.7 | 59 | 6.0 | 61 | 7.6 |  |
| Injury intent | |  |  |  |  |  |  | 0.257 |
|  | Accidental | 1741 | 97.8 | 957 | 97.5 | 784 | 98.3 |  |
|  | Intentional | 39 | 2.2 | 25 | 2.6 | 14 | 1.8 |  |
| Mechanism of injury | |  |  |  |  |  |  | <0.001 |
|  | Traffic accident | 792 | 44.5 | 490 | 49.9 | 302 | 37.8 |  |
|  | Fall | 773 | 43.4 | 394 | 40.1 | 379 | 47.5 |  |
|  | Collision | 173 | 9.7 | 74 | 7.5 | 99 | 12.4 |  |
|  | Others | 42 | 2.4 | 24 | 2.4 | 18 | 2.3 |  |
| Place of injury | |  |  |  |  |  |  | 0.123 |
|  | Home | 481 | 27.0 | 251 | 25.6 | 230 | 28.8 |  |
|  | Public | 1299 | 73.0 | 731 | 74.4 | 568 | 71.2 |  |
| Alcohol intake | |  |  |  |  |  |  | <0.001 |
|  | Yes | 1371 | 77.0 | 726 | 73.9 | 645 | 80.8 |  |
|  | No | 409 | 23.0 | 256 | 26.1 | 153 | 19.2 |  |
| Day of injury | |  |  |  |  |  |  | 0.826 |
|  | Weekend | 549 | 30.8 | 305 | 31.1 | 244 | 30.6 |  |
|  | Weekday | 1231 | 69.2 | 677 | 68.9 | 554 | 69.4 |  |
| Time of injury | |  |  |  |  |  |  | 0.471 |
|  | Day (7.00 AM - 6.59 PM) | 1025 | 57.6 | 558 | 56.8 | 467 | 58.5 |  |
|  | Night (7.00 PM - 6.59 AM) | 755 | 42.4 | 424 | 43.2 | 331 | 41.5 |  |
| Top-level of personnel | |  |  |  |  |  |  | <0.001 |
|  | Physician | 77 | 4.3 | 7 | 0.7 | 70 | 8.8 |  |
|  | Nurse | 353 | 19.8 | 280 | 28.5 | 73 | 9.2 |  |
|  | EMT | 1084 | 60.9 | 613 | 62.4 | 471 | 59.0 |  |
|  | First responder | 122 | 6.9 | 68 | 6.9 | 54 | 6.8 |  |
|  | Unknown | 144 | 8.1 | 14 | 1.4 | 130 | 16.3 |  |
| Airway management | |  |  |  |  |  |  | <0.001 |
|  | Advanced airway | 27 | 1.5 | 26 | 2.7 | 1 | 0.1 |  |
|  | Basic airway | 72 | 4.0 | 72 | 7.3 | 0 | 0.0 |  |
|  | No airway management | 830 | 46.6 | 829 | 84.4 | 1 | 0.1 |  |
|  | Unknown | 851 | 47.8 | 55 | 5.6 | 796 | 99.8 |  |
| Ventilatory management | |  |  |  |  |  |  | <0.001 |
|  | Active ventilatory support | 68 | 3.8 | 67 | 6.8 | 1 | 0.1 |  |
|  | Passive ventilatory support | 358 | 20.1 | 358 | 36.5 | 0 | 0.0 |  |
|  | No ventilatory support | 503 | 28.3 | 502 | 51.1 | 1 | 0.1 |  |
|  | Unknown | 851 | 47.8 | 55 | 5.6 | 796 | 99.8 |  |
| Intravenous fluid | |  |  |  |  |  |  | <0.001 |
|  | Yes | 148 | 8.3 | 147 | 15.0 | 1 | 0.1 |  |
|  | No | 781 | 43.9 | 780 | 79.4 | 1 | 0.1 |  |
|  | Unknown | 851 | 47.8 | 55 | 5.6 | 796 | 99.8 |  |
| Systolic blood pressure, mmHg | |  |  |  |  |  |  | 0.121 |
|  | < 90 | 104 | 5.8 | 65 | 6.6 | 39 | 4.9 |  |
|  | ≥ 90 | 1676 | 94.2 | 917 | 93.4 | 759 | 95.1 |  |
| Heart rate, beats per minute | |  |  |  |  |  |  | 0.029 |
|  | < 60 | 122 | 6.9 | 76 | 7.7 | 46 | 5.8 |  |
|  | 60-100 | 1238 | 69.6 | 658 | 67.0 | 580 | 72.7 |  |
|  | > 100 | 420 | 23.6 | 248 | 25.3 | 172 | 21.6 |  |
| Respiratory rate, rate per minute | |  |  |  |  |  |  | 0.013 |
|  | < 10 | 9 | 0.5 | 4 | 0.4 | 5 | 0.6 |  |
|  | 10-30 | 1728 | 97.1 | 945 | 96.2 | 783 | 98.1 |  |
|  | > 30 | 43 | 2.4 | 33 | 3.4 | 10 | 1.3 |  |
| Glasgow coma scale | |  |  |  |  |  |  | <0.001 |
|  | 3-8 | 657 | 36.9 | 443 | 45.1 | 214 | 26.8 |  |
|  | 9-12 | 292 | 16.4 | 164 | 16.7 | 128 | 16.0 |  |
|  | 13-15 | 663 | 37.3 | 331 | 33.7 | 332 | 41.6 |  |
|  | Unknown | 168 | 9.4 | 44 | 4.5 | 124 | 15.5 |  |
| Intervention | |  |  |  |  |  |  |  |
|  | Neurosurgical | 1754 | 98.5 | 960 | 97.8 | 794 | 99.5 | 0.002 |
|  | Neuroradiological | 40 | 2.3 | 31 | 3.2 | 9 | 1.1 | 0.004 |
| Time to intervention | |  |  |  |  |  |  | <0.001 |
|  | Early (<1.9 h) | 532 | 29.9 | 258 | 26.3 | 274 | 34.3 |  |
|  | Intermediate (1.9-4.1 h) | 541 | 30.4 | 304 | 31.0 | 237 | 29.7 |  |
|  | Delayed (>4.1 h) | 707 | 39.7 | 420 | 42.8 | 287 | 36.0 |  |
| EMR-ISS | |  |  |  |  |  |  | 0.002 |
|  | 0-24 | 93 | 5.22 | 67 | 6.82 | 26 | 3.26 |  |
|  | 25-44 | 1472 | 82.7 | 790 | 80.5 | 682 | 85.5 |  |
|  | 45-75 | 215 | 12.1 | 125 | 12.7 | 90 | 11.3 |  |
| Outcomes | |  |  |  |  |  |  |  |
|  | Death | 455 | 25.6 | 289 | 29.4 | 166 | 20.8 | <0.001 |
|  | Unfavorable neurological outcome | 944 | 53.0 | 529 | 53.9 | 415 | 52.0 | 0.241 |

EMR-ISS, excess mortality ratio-adjusted injury severity score

Table S2 Interaction effect of time to neurosurgical and neuroradiological intervention with the mode of transport on outcomes

| Outcomes | Time to intervention | Mode of transport | | | | | |
| --- | --- | --- | --- | --- | --- | --- | --- |
|  |  | Prehospital transport | | | Interhospital transport | | |
|  |  | AOR | 95% CI | | AOR | 95% CI | |
| Death |  |  |  | |  |  | |
|  | Early | 1.00 |  |  | 1.00 |  |  |
|  | Intermediate | 1.27 | 0.75 | 2.14 | 0.84 | 0.70 | 1.00* |
|  | Delayed | 1.06 | 0.64 | 1.73 | 0.93 | 0.77 | 1.12 |
| Unfavorable neurological outcome | |  |  |  |  |  |  |
|  | Early | 1.00 |  |  | 1.00 |  |  |
|  | Intermediate | 0.89 | 0.55 | 1.43 | 0.89 | 0.76 | 1.05 |
|  | Delayed | 0.88 | 0.57 | 1.36 | 0.97 | 0.82 | 1.14 |

AOR, adjusted odds ratio

CI, confidence interval

*statistically significant

The interaction model was adjusted for age, gender, Charlson comorbidity index, injury intent, mechanism of injury, place of injury, alcohol intake, time of injury, top-level personnel, intravenous fluid, systolic blood pressure, heart rate, Glasgow coma scale score, and EMR-ISS.
